# RNA sequencing analyses reveal novel differentially expressed genes and pathways in pancreatic cancer

**DOI:** 10.18632/oncotarget.16451

**Published:** 2017-03-22

**Authors:** Yixiang Mao, Jianjun Shen, Yue Lu, Kevin Lin, Huamin Wang, Yanan Li, Ping Chang, Mary G. Walker, Donghui Li

**Affiliations:** ^1^ Department of Oncology, The First Affiliated Hospital of Soochow University, Suzhou 215007, China; ^2^ Department of Gastrointestinal Medical Oncology, The University of Texas MD Anderson Cancer Center, Houston, Texas 77030, USA; ^3^ Department of Pathology and Department of Translational Molecular Pathology, The University of Texas MD Anderson Cancer Center, Houston, Texas 77030, USA; ^4^ Department of Epigenetics and Molecular Carcinogenesis, The University of Texas MD Anderson Cancer Center, Smithville, Texas 78957, USA; ^5^ Department of Medical Oncology, Fudan University Shanghai Cancer Center, Shanghai 200032, China

**Keywords:** pancreatic cancer, RNA sequencing, transcriptome, pathway analysis

## Abstract

Gene expression microarrays have identified many tumor markers and therapeutic targets for pancreatic ductal adenocarcinoma (PDAC). However, microarray profilings have limited sensitivity and are prone to cross-hybridization between homologous DNA fragments. Here, we perform a transcriptome analysis of paired tumor and adjacent benign pancreatic tissues from 10 patients who underwent resection for PDAC. We identify a total of 2736 differentially expressed genes (DEGs) with false discovery rate less than 0.05, including 1554 upregulated, 1182 downregulated, and 6 microRNAs (miR-614, miR-217, miR-27b, miR-4451, miR-3609, and miR-612). Overexpression of five DEGs, i.e. *KRT16*, *HOXA10*, *CDX1*, *SI*, and *SERPINB5* in tumors is confirmed by RT-PCR in 20 additional tissues. Overexpression of *KRT16* in PDAC is also verified on protein level. In addition, top canonical pathways such as granulocyte adhesion and diapedesis pathway have been identified. Our study represents a comprehensive characterization of the PDAC transcriptome and provides insight to the mechanisms of pancreatic carcinogenesis and potential biomarkers and novel therapeutic targets for pancreatic cancer.

## INTRODUCTION

Pancreatic ductal adenocarcinoma (PDAC) is a highly lethal disease with a 5-year survival rate of 6% [1]. PDAC is usually diagnosed at late stage which prelude the chance of tumor resection for cure. PDAC is also highly aggressive and resistant to most therapies. Previous studies of large-scale gene expression analysis have used the microarray approach to identify novel tumor markers and potential therapeutic targets for PDAC [2]. However, microarray analyses have limited sensitivity and are prone to cross-hybridization between homologous DNA fragments [3]. With the advancement of the next-generation sequencing technologies, RNA sequencing (RNA-seq) has become a useful tool in defining the transcriptomes of cells. Compared to microarray analysis, RNA-seq has the advantage of higher sensitivity and the ability to detect splicing isoforms and somatic mutations [4, 5]. A few studies have been conducted in pancreatic cancer using RNA-seq method, but most of these studies were conducted in cell lines [6] and circulating tumor cells [7, 8]. Gene expression profiling in PDAC tissue samples using the microarray approach were mostly conducted in patients with PDAC versus patients without cancer [9–12] or in tissue samples from PDAC patients with different clinical or pathological features [13–16]. Literature search failed to find any transcriptome analysis in comparing the tumor and adjacent benign pancreatic tissues in pancreatic cancer. To fill in this gap, we embarked on a study using RNA-seq to compare the transcriptomes of 10 paired tumor and adjacent benign pancreatic tissue samples from patients who underwent resection for PDAC. Novel differentially expressed genes and canonical pathways were identified by this approach, which may open new research venue for pancreatic cancer.

## RESULTS

### RNA-seq

RNA-seq was successfully carried out in all 20 samples. All sequence data were read at a length of 2×76 bp with high-quality metrics (>28 Phred score) and nucleotide distributions. The total number of sequenced reads ranged from 25 million to 33 million pairs, and an average 95.5% (range: 92.2%-97.6%) of the pairs were aligned to the hg19 genome assembly using the TopHat2 aligner. The percentage of genomic alignment was similar between the tumor and non-tumor tissues (mean ± standard deviation: 96.1±1.1% and 95.0±1.8%, respectively), suggesting no obvious detectable biases in the sequence data (*P* = 0.11). Alignment statistics indicated the data were of high quality and were uniform (i.e., no outliers with reference to alignment proficiency) and provided sufficient sequencing depth to pursue differential expression testing between two groups.

### Estimated purity of the tissue samples

The purity of tumor and adjacent non-tumor tissue used in RNA–seq was 0.73 ± 0.10 and 0.80 ± 0.08, respectively as predicted by the “Estimation of STromal and Immune cells in MAlignant Tumours using Expression data” (ESTIMATE) method (paired t-test, *P* = 0.10). There was no significant correlation between these two groups (r = 0.124, *P* = 0.73).

### Identification of DEGs

**Figure 1:**
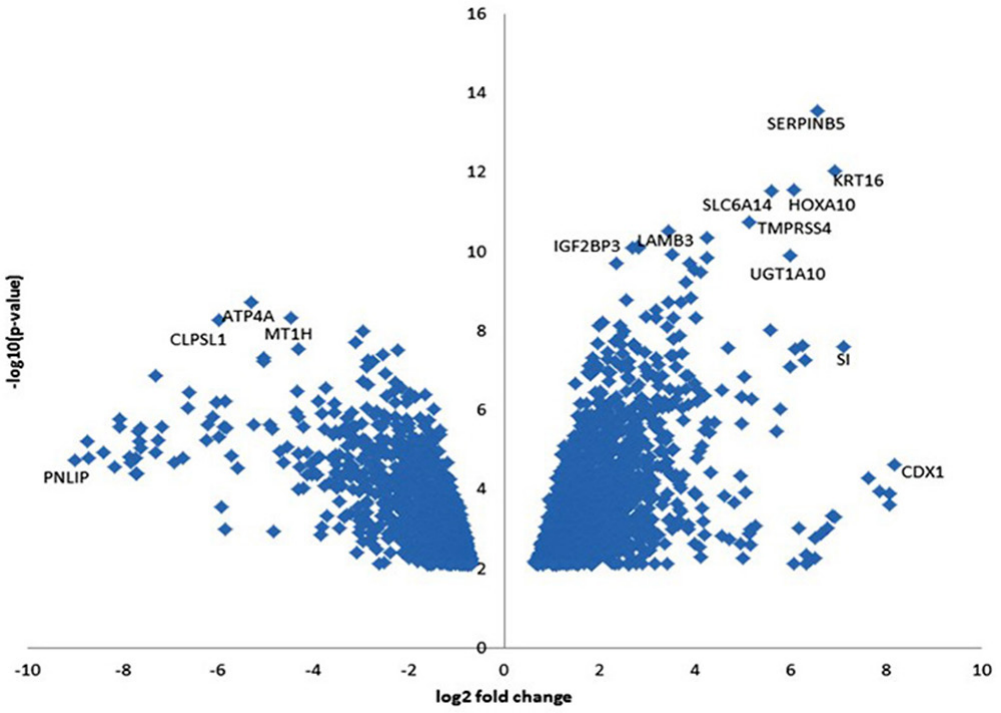
Volcano plot of DEGs (PDR < 0.05) in tumor and adjacent benign pancreatic tissues The horizontal axis is the log2 fold change between PDAC and adjacent benign pancreatic tissues. The negative log10 of the *P*-value of Fisher's exact test is plotted on the vertical axis. Each gene is represented by one point on the graph.

We identified 2736 DEGs with false discovery rate (FDR)<0.05 including 1554 upregulated and 1182 downregulated genes (Supplementary Table 3). Although RNA-seq was trimmed to detect mRNA, we found that 6 microRNAs were enriched in the DEGs: two were upregulated (miR-614 and miR-612), and four were downregulated (miR-217, miR-27b, miR-4451, and miR-3609) (Table 1). To select DEGs, we ranked genes by the log10 *P* value of genes with FDR (q-value) < 0.05 and plotted them against the log2 fold change in a “volcano” plot (Figure 1). We identified 17 genes that were upregulated and 36 genes that were downregulated with FDR (q-value) <0.001 and log ratio ≥5 (Table 1). Among the 17 overexpressed genes, *CDX1 (caudal type homeobox 1)* had the highest fold difference in tumor versus non-tumor tissues followed by *SI (sucrase-isomaltase, aka alpha-glucosidase*), *KRT16 (keratin 16) and SERPINB5* (*serpin peptidase inhibitor, clade B (ovalbumin), member 5*). *SERPINB5* followed by *KRT16* and *HOXA10* had the smallest *P* values and FDR q-values. The 36 downregulated genes included many genes coding for digestive enzymes, which reflect the impairments of exocrine pancreatic functions by the tumor.

**Table 1:** Top differentially expressed genes (FDR<0.001 and log2ratio≥5) and miRNAs

Symbol	Gene Name	Log2Ratio	*P*-value	FDR (q-value)
**Upregulated**				
*CDX1*	caudal type homeobox 1	8.166	2.28×10^−5^	7.83×10^−4^
*SI*	sucrase-isomaltase (alpha-glucosidase)	7.111	2.42×10^−8^	8.73×10^−6^
*KRT16*	keratin 16	6.917	9.18×10^−13^	7.94×10^−9^
*SERPINB5*	serpin peptidase inhibitor, clade B (ovalbumin), member 5	6.561	2.80×10^−14^	4.85×10^−10^
*TINAG*	tubulointerstitial nephritis antigen	6.286	5.46×10^−8^	1.43×10^−5^
*CST1*	cystatin SN	6.250	2.27×10^−8^	8.36×10^−6^
*PITX1*	paired-like homeodomain 1	6.081	2.78×10^−8^	9.47×10^−6^
*HOXA10*	homeobox A10	6.054	2.70×10^−12^	1.28×10^−8^
*LINC00460*	long intergenic non-protein coding RNA 460	5.987	7.85×10^−8^	1.76×10^−5^
*UGT1A9*	UDP glucuronosyltransferase 1 family, polypeptide A8	5.978	1.23×10^−10^	1.78×10^−7^
*SLCO1B7*	solute carrier organic anion transporter family, member 1B7	5.772	9.51×10^−7^	9.84×10^−5^
*HOTTIP*	HOXA distal transcript antisense RNA	5.707	3.37×10^−6^	2.19×10^−4^
*SLC6A14*	solute carrier family 6 (amino acid transporter), member 14	5.586	2.96×10^−12^	1.28×10^−8^
*CEACAM5*	carcinoembryonic antigen-related cell adhesion molecule 5	5.582	9.32×10^−9^	4.61×10^−6^
*MSLN*	mesothelin	5.187	5.31×10^−7^	6.81×10^−5^
*TMPRSS4*	transmembrane protease, serine 4	5.118	1.80×10^−11^	6.23×10^−8^
*SLCO1B3*	solute carrier organic anion transporter family, member 1B3	5.031	1.47×10^−7^	2.62×10^−5^
Downregulated*				
*SYCN*	syncollin	−8.059	1.70×10^−6^	1.44×10^−4^
*PLA2G1B*	phospholipase A2, group IB (pancreas)	−7.822	2.01×10^−5^	7.17×10^−4^
*GP2*	glycoprotein 2 (zymogen granule membrane)	−7.728	1.63×10^−5^	6.14×10^−4^
*RBPJL*	recombination signal binding protein for immunoglobulin kappa J region-like	−6.646	8.63×10^−7^	9.24×10^−5^
*SERPINI2*	serpin peptidase inhibitor, clade I (pancpin), member 2	−6.620	3.63×10^−7^	5.18×10^−5^
*PRSS3*	protease, serine, 3	−6.240	5.50×10^−6^	3.05×10^−4^
*KLK1*	kallikrein 1	−6.224	2.31×10^−6^	1.74×10^−4^
*ERP27*	endoplasmic reticulum protein 27	−6.103	1.51×10^−6^	1.34×10^−4^
*PDIA2*	protein disulfide isomerase family A, member 2	−6.044	6.12×10^−7^	7.51×10^−5^
*AQP8(Ins)*	aquaporin 8	−5.982	4.63×10^−6^	2.76×10^−4^
*CLPSL1*	colipase-like 1	−5.977	5.12×10^−9^	2.95×10^−6^
*GPHA2*	glycoprotein hormone alpha 2	−5.868	2.58×10^−6^	1.86×10^−4^
*GUCA1C*	guanylate cyclase activator 1C	−5.841	5.92×10^−7^	7.42×10^−5^
*GSTA2*	glutathione S-transferase alpha 2	−5.827	2.73×10^−6^	1.91×10^−4^
*TMEM52*	transmembrane protein 52	−5.711	1.38×10^−5^	5.52×10^−4^
*PNLIPRP2*	pancreatic lipase-related protein 2	−5.596	2.95×10^−5^	9.39×10^−4^
*ATP4A*	ATPase, H+/K+ exchanging, alpha polypeptide	−5.319	1.82×10^−9^	1.44×10^−6^
*PM20D1*	peptidase M20 domain containing 1	−5.251	2.34×10^−6^	1.74×10^−4^
*C12orf39*	chromosome 12 open reading frame 39	−5.045	4.83×10^−8^	1.33×10^−5^
*CCKBR(Ins)*	cholecystokinin B receptor	−5.041	5.81×10^−8^	1.44×10^−5^
**MiRNA**				
miR-614		3.64	3.36×10^−7^	4.92×10^−5^
miR-217		−4.21	2.64×10^−6^	1.87×10^−4^
miR-27b		−2.15	3.10×10^−4^	0.0048
miR-4451		−1.74	1.09×10^−3^	0.012
miR-3609		−1.15	1.57×10^−3^	0.015
miR-612		0.91	4.81×10^−3^	0.034

^*^ Sixteen downregulated genes that are coding for pancreatic digestive enzymes are not listed. The complete list of the downregulated genes are presented in Supplementary Table 3.

### Validation analysis using quantitative RT-PCR and IHC

**Figure 2:**
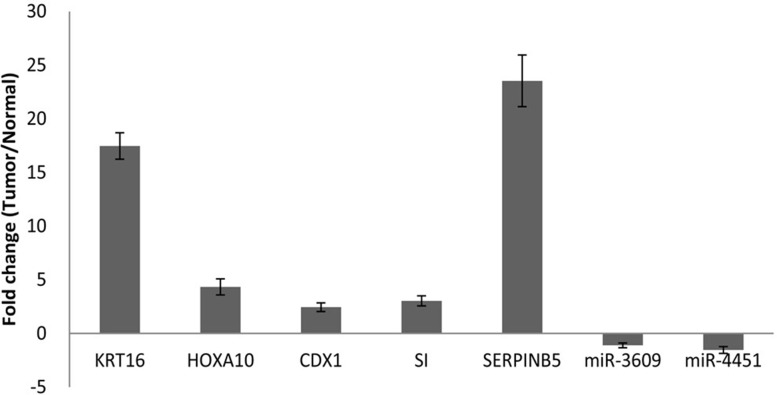
qRT-PCR analysis of KRT16, HOXA10, CDX1, SI, SERPINB5, miR-3609, and miR-4451 in PDAC Real-time quantitative PCR was performed with gene-specific primers. The expression of each gene was normalized with the average expression of the endogenous reference gene β-actin. The logarithm of relative quantitation in the gene expression of corresponding transcripts in 20 tumor tissues compared to 20 adjacent non-tumor tissues is plotted in the graph. The error bar indicates the standard error of the mean fold change.

**Figure 3:**
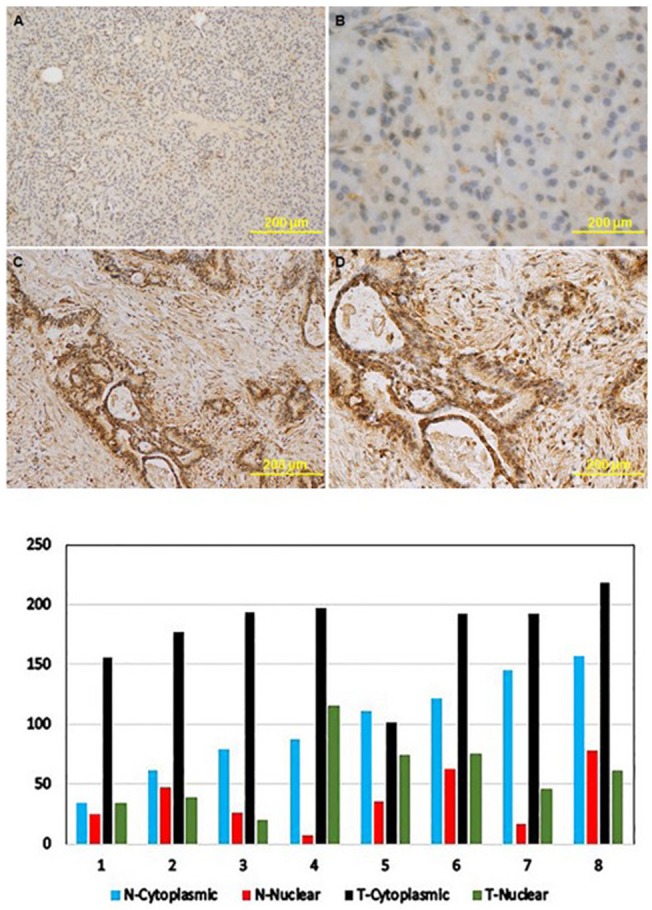
The expression levels of KRT16 protein in paired tumor and benign pancreatic tissues from patients who underwent resection for PDAC Upper panels: immunohistochemistry images: **(A)** and **(B)**, KRT16 expression in normal pancreatic tissues; **(C)** and **(D)**, KRT16 expression in PDAC. Magnification was x40 for panel **(A)** and **(C)** and x100 for panel **(B)** and **(D)**. Lower pane: KRT16 staining scores in PDAC (T) and benign pancreatic tissues (N).

Among the 17 upregulated genes, we selected the top five, i.e. *CDX1, SI*, *KRT16, HOXA10*, and *SERPINB5* for validation using RT-PCR in the 20 pairs of tumor and non-tumor tissues that were not used in RNA-seq. The RT-PCR results confirmed overexpression of all five genes in pancreatic tumors compared to non-tumor tissues (Figure 2). The largest fold difference in mRNA expression between tumor and non-tumor tissues was seen for *SERPINB5* and *KRT16*. Because KRT16 protein expression has not been previously investigated in pancreatic cancer, we further conducted immunohistochemistry (IHC) to compare the expression level of KRT16 protein in eight pairs of tumor and adjacent non-tumor tissues from patients who underwent resection for PDAC. KRT16 staining was present in both cytoplasm and nucleus of the normal ductal epithelia (Figure 3, upper panels) and tumor cells (Figure 3, lower panel). But the protein expression was mostly detected in the cytoplasm. Tumor tissues showed a significantly higher level of KRT16 expression than non-tumor tissues, especially in cytoplasm. The average H-score for KRT16 expression was 236.1 ± 46.8 in tumors and 135.8 ± 56.8 in non-tumor tissues, respectively (*P* = 0.002).

### IPA analyses of DEGs

Ingenuity Pathway Analysis (IPA; Ingenuity Systems/Qiagen, Redwood City, CA, USA) of DEGs with a FDR q-value of <0.01 revealed 99 significant canonical pathways (Supplementary Table 4) and 21 significant molecular and cellular functions (Supplementary Table 5) (Fisher's exact test, *P* < 0.05). The top five canonical pathways are the granulocyte adhesion and diapedesis, inhibition of matrix metalloproteases, lipopolysaccharide/interleukin-1-mediated inhibition of retinoid X receptor function, antigen presentation, and complement system pathways. The major contribution genes to each of the five pathways are listed in Table 2. The five top cellular functions that are over-represented by DEGs are cellular growth and proliferation, cellular movement, cell death and survival, cell to cell signaling and interactions, and cellular development (Supplementary Table 5).

**Table 2:** Top canonical pathways and molecular functions enriched by DEGs*

Canonical pathways	*P*-value	Ratio	Molecules
Granulocyte Adhesion and Diapedesis	8.56×10^−8^	33/177 (0.186)	*FPR3,IL1A,MMP3,MMP14,MMP13,CCL20,CCL22,CXCL5,IL1R2,CXCL10,HRH1,CCL13,CXCL13,CCL28,MMP11,CXCL17,MMP12,TNFRSF1B,MMP1,TNFRSF11B,CLDN10,SDC1,MMP28,ITGA2,MMP10,ITGAL,SELPLG,C5,ITGB2,ITGAM,IL1RN,CCL18,MMP9*
Inhibition of Matrix Metalloproteases	5.95×10^−6^	12/39 (0.308)	*SDC1,MMP28,MMP3,TIMP1,MMP14,MMP10,MMP13,MMP11,MMP12,MMP9,LRP1,MMP1*
LPS/IL-1 Mediated Inhibition of RXR Function	1.16×10^−5^	33/219 (0.151)	*IL1A,CHST4,CYP2C9,ABCG1,CYP2C19,IL1R2,ALDH1A1,UST,NR0B2,NR1I2,ALDH3A2,ACSL5,HS6ST2,CHST11,HS3ST1,HS6ST3,GSTA1,LBP,TNFRSF1B,SLCO1B3,ALDH6A1,TNFRSF11B,GSTA2,GSTA4,IL4I1,TLR4,FABP2,SULT1E1,ALDH1L2,IL1RN,NR5A2,GSTO2,SULT1B1*
Antigen Presentation Pathway	2.11×10^−5^	11/37 (0.297)	*PSMB9,NLRC5,HLA-A,HLA-DMB,CIITA,HLA-DOB,PSMB8,HLA-F,TAP1,TAP2,TAPBP*
Complement System	2.11×10^−5^	11/37 (0.297)	*ITGB2,CR1,ITGAM,C4BPB,CFB,C1QC,C6,C1QB,C2,CR2,C5*
Leukocyte Extravasation Signaling	2.56×10^−5^	30/198 (0.152)	*RAC2,MMP3,PTK2B,MMP14,MMP13,RHOH,NOX1,TIMP1,CYBB,MMP11,MMP12,MMP1,ACTN1,CLDN10,PIK3C2B,MMP28,ITGA2,MMP10,ITGAL,SELPLG,ITGB2,WIPF1,ITGAM,EDIL3,RAP1GAP,NCF2,PIK3R6,VAV1,MMP9,PRKCB*

^*^ Canonical pathway analysis identified the pathways from the IPA library of canonical pathways that were most significant enriched in differentially expressed genes. Genes with FDR q-value of <0.01 from data set that were associated with a canonical pathway in the Ingenuity Knowledge Base were considered for the analysis. The significance of the association between the data set and the pathway was measured in 2 ways: 1) a ratio of the number of molecules from the data set that map to the pathway divided by the total number of molecules that map to the canonical pathways is displayed. 2) Fisher's exact test was used to calculate a p-value determining the probability that the association between the genes in the observed values and the canonical pathway is explained by chance alone.

### Upstream transcriptors enriched by DEGs

The 15 most significantly activated or inhibited upstream transcription regulators identified by IPA are listed in Table 3. Among the inhibited upstream transcriptors are important tumor suppressor genes, such as *TP53, CDKN2A* and *RB1*. On the other hand, the activated upstream regulators mostly are signal transducers that play critical roles in inflammatory or immune response and tumorigenesis, e.g. *STAT3, CTNNB1, SP1*, and *NFκB etc*. Notably, two pancreatic cancer susceptibility genes previously identified by genome wide association studies, i.e. *NR5A2* (nuclear receptor group 5A member 2) [17] and *HNF1A* (hepatocyte nuclear factor 1 homeobox A) [18], were among the inhibited upstream transcription regulators.

**Table 3:** Top 15 significant upstream transcription regulators

Upstream Regulator	Log Ratio	Activation z-score^#^	*P*-value of overlap^&^
**Inhibition**			
*TP53*		−2.353	3.34×10^−32^
*NUPR1*	−2.52	−3.977	4.62×10^−13^
*NKX2-3*		−3.455	5.88×10^−12^
*HNF1A*		−2.359	8.82×10^−12^
*CDKN2A*	1.737	−3.219	1.34×10^−11^
*estrogen receptor*		−2.299	9.57×10^−10^
*RB1*		−4.286	3.74×10^−08^
*TCF3*		−3.116	4.32×10^−08^
*TRIM24*		−3.714	6.26×10^−07^
*BCL6*		−2.313	1.60×10^−06^
*NR5A2*	−2.876	−2.7	7.26×10^−06^
*SATB1*	−0.814	−2.003	1.09×10^−05^
*IRF4*	2.115	−2.41	4.24×10^−05^
*RBL1*		−3.124	1.72×10^−04^
*SPDEF*		−2.887	2.39×10^−04^
**Activation**			
*STAT3*		2.924	5.78×10^−19^
*CTNNB1*		3.359	1.99×10^−15^
*SP1*		2.06	4.79×10^−13^
*CEBPB*		2.866	2.62×10^−12^
*NFkB (complex)*		6.314	1.43×10^−11^
*TBX2*		4.99	1.62×10^−11^
*IRF1*		3.759	2.76×10^−11^
*IRF7*	1.504	5.591	2.88×10^−10^
*FOXM1*	2.152	4.395	5.12×10^−10^
*STAT1*		4.576	2.05×10^−08^
*ETS1*	0.863	2.575	5.06×10^−08^
*JUN*		2.045	5.57×10^−08^
*FOXO1*		3.242	9.35×10^−08^
*E2F3*	1.137	2.394	1.16×10^−06^
*MBD2*		2.549	5.41×10^−06^

^#^Activation z-score is a statistical parameter that determines whether an upstream transcription regulator has significantly more “activated” predictions than “inhibited” predictions (*z*>0) or vice versa (*z*<0). Here, significance means that we reject the hypothesis that predictions are random with equal probability.

^&^Overlap *P*-value measures whether there is a statistically significant overlap between the dataset genes and the genes that are regulated by a transcriptional regulator. It is calculated using Fisher's Exact Test, and significance is generally attributed to *P*-values < 0.01. Since the regulation direction (“activating” or “inhibiting”) of an edge is not taken into account for the computation of overlap *P*-values the underlying network also includes findings without associated directional attribute, such as protein-DNA (promoter) binding.

## DISCUSSION

To our knowledge, this is the first report of a comprehensive transcriptome analysis using RNA-seq in pancreatic cancer. In 10 pairs of PDAC tumor and adjacent benign pancreatic tissues, a large number (2,736) of DEGs were identified. Validation of overexpression of the top five DEGs at the RNA or protein levels suggest their potential values as biomarker or therapeutic targets in pancreatic cancer. IPA analysis has revealed several canonical pathways and molecular functions that are associated with pancreatic cancer. These findings opened new research venues for pancreatic cancer.

Using the RNA-seq technique, we identified much more DEGs in the current study compared with previous expression profiling analysis that used the microarray approach. RNA-seq is a more sensitive technology than expression profiling analysis using arrays, which is limited by its low sensitivity due to background hybridization and sometimes reduced specificity due to cross-hybridization of probes and targets [19, 20]. Comprehensive characterization of the transcriptome of PDAC is critical to understanding the disease at a system-wide level, as any missing data would create a biased view of this deadly disease.

Among the five top DEGs, overexpression of *SERPINB5* [9, 11, 21–23], *HOXA10* [12, 24] and KRT16 [9, 10, 15] at the mRNA level has previously been reported in pancreatic cancer. *SERPINB5* expression has been associated with clinical outcome of several types of human cancers [25–27]. *HOXA10* is a DNA-binding transcription factor that may regulate gene expression, morphogenesis, and differentiation. Keratin 16 expression is regulated by epithelial growth factor [28] and it regulates innate immune functions [29]. A higher expression of *KRT16* was observed in tumor than its adjacent normal pancreatic tissue in our study. Overexpression of *KRT16* mRNA has been identified as a prognostic markers in triple negative breast cancer [30]. Findings from the RNA-seq, RT-PCR and IHC experiments in the current study provide additional support for their potential role in pancreatic cancer. CDX1 has been shown to inhibit beta-catenin/T-cell factor transcriptional activity [31]. SI plays a critical role in the digestion of dietary carbohydrates including starch, sucrose and isomaltose [32]. Along with six other top DEGs, i.e. *TINAG, LINC00460, UGT1A9, SLCO1B7, HOTTIP*, and *ALCO1B3*, their expression status could not be found in the Pancreatic Cancer Database [33]. Among the top 34 downregulated genes, *SYCN, RBPJL, CLPSL1, GPHA2, GUCA1C GSTA2, TMEM52, ATP4A, PM20D1*, and *C12orf39* have not previously been reported in pancreatic cancer. The role of these DEGs in pancreatic cancer needs further investigation.

IPA analyses indicated that the DEGs were mostly enriched in 21 significant molecular and cellular functions and 99 significant canonical pathways, which provides important clues for understanding the molecular mechanisms of PDAC pathogenesis. The overlapping networks of pathways were closely related to inflammatory and immune response, regulation of the cell cycle, and nicotine and neurotransmitter degradation. The major cellular functions of the DEGs represented include the cellular growth and proliferation, cellular movement, cell death and survival, cell to cell signaling and interactions, and cellular development. In contrast to a previous report on loss of expression of antigen-presenting molecules in human PDAC and PDAC cell lines [34], we observed upregulation of many antigen presentation-related genes in PDAC tissues. This discrepancy can be explained by the fact that the previous study compared PDAC tissue samples with the benign pancreatic samples from patients with benign pancreatic disease and downregulation of expression of antigen processing and antigen-presenting molecules reflected tumor evading immune recognition and destruction. The current study compared tumor with adjacent benign pancreatic tissues from the same PDAC patients. The upregulation of antigen presenting molecules reflect an inflammatory feature of the PDAC [35, 36].

Interestingly, although the RNA-seq was trimmed to detect mRNA, we found that 6 microRNAs in the DEGs, i.e. miR-614 and miR-612 were upregulated, miR-217, miR-27b, miR-4451, and miR-3609 were downregulated in PDAC tissues compared with adjacent tissues. IPA showed that miR-3609 and miR-4451 were related to PIGG mRNA which is involved in the biosynthesis of glycosylphosphatidylinositol anchor. The role of these 2 downregulated microRNAs in PDAC remains to be studied.

Taken together, the results of our RNA-Seq analysis suggest that malignant transformation of pancreatic ductal cells involves the perturbation of multiple important cellular pathways, including cell growth-related pathways, metabolism-related processes, and immune-related and miRNA-regulated pathways.

Tissue cellularity is always a great challenge in PDAC research because PDAC consists of a higher percentage of stromal cells than other solid tumors. The infiltrating stromal and immune cells form the major fraction of normal cells in tumor tissue and may interfere with the tumor signal in molecular studies. In the current study, we restricted our tissue samples for RNA-seq to those with >70% tumor cells in the tumor samples. We also used the ESTIMATE method [37] in which gene expression signatures are used to infer the fraction of stromal and immune cells in tumor samples. The average tumor purity prediction of 2,463 samples using ESTIMATE signatures was 0.61±0.20. Although second-generation sequencing platforms facilitate the use of more heterogeneous samples, they may still underestimate the differential expression between cancer and normal tissues. Future work using microdissected tumor cells will help increase the accuracy of prediction.

In conclusion, this study was the first to use the RNA-Seq platform to comprehensively characterize the PDAC transcriptome. We identified a number of genes that were dysregulated in PDAC and may serve as targets for biomarker evaluation and therapeutic intervention. Follow-up analysis of modulator genes found in this study might be useful for acquiring a deeper understanding of pathological changes in PDAC and for developing prospective diagnostic and intervention strategies.

## EXPERIMENTAL PROCEDURES

### Tissue samples

Paired tumor and adjacent benign pancreatic tissues were obtained from 30 patients with PDAC who underwent tumor resection at the Fudan University Cancer Hospital in Shanghai, China, from May 2010 to February 2012. Information on patients’ demographics, tumor location, histopathologic tumor type, histologic grade, tumor stage, lymph node metastasis, serum CA19-9 level, and performance status were collected from medical records. The characteristics of the 30 patients are summarized in Supplementary Table 1. No patients had received preoperative therapy. Informed consent was obtained from all patients, and the study was approved by the Institute Research Ethics Committee at Fudan University. Fresh samples of tumor and adjacent benign pancreas from each patient were harvested immediately after the surgery, washed with sterile normal saline, frozen in RNAlater (ThermoFisher Scientific, Grand Island, NY) in liquid nitrogen overnight and then transferred to a -80°C freezer. Tumor and adjacent benign pancreatic tissues samples were confirmed via histopathologic examination by frozen sections. Briefly, the samples of tumor and adjacent benign pancreatic tissue were frozen in optimum cutting temperature compound for sectioning. A 5 μM sections were prepared from each sample for hematoxylin and eosin staining. The cellularity of tumor sections was determined microscopically by a pathologist. Sections from 10 patients with a cellularity of greater than 70% and no necrosis were selected for RNA-seq. The remaining 20 samples with cellularity ranging from 15% to 65% were used for validation experiments.

### RNA-Seq

Total RNA was isolated from frozen tissue blocks containing about 50-100 mg tissues using TRI Reagent (Molecular Research Center Inc., OH) following the manufacturer's instructions. The quality, quantity, and integrity of the total RNA were evaluated using a NanoDrop1000 spectrophotometer and Bioanalyzer 2100 (Agilent Technologies, CA). Samples with a RNA quality (RIN) score of >7.0 was used in RNA-seq. A mRNA-focused, barcoded library was generated using TruSeq RNA Sample Preparation Kits (Illumina, CA) with the ovation RNA-Seq System V2 (NuGEN Technologies, Inc., San Carlos, CA). The libraries were sequenced on an Illumina HiSeq 2000 instrument (San Diego, CA) with 2×76-base pair (bp) paired end protocol at the Science Park NGS Facility. Totally 20 libraries (paired tumor and adjacent benign tissues) from 10 patients with resected PDAC were sequenced, generating 25-33 million pairs of reads per sample. Each pair of reads represents a cDNA fragment from the library

The quality of the sequencing data was analyzed by the bioinformatics team associated with the Science Park NGS Facility using FastQC (http://www.bioinformatics.babraham.ac.uk/projects/fastqc/). The reads were mapped to human genome (hg19) by TopHat (version 2.0.4) [38] and Bowtie2 (version 2.0.0-beta7) [39]. 92.2-97.6% fragments were mapped to human genome. The number of fragments in each known gene from RefSeq database [40] (downloaded from UCSC Genome Browser on March 6, 2013) was enumerated using htseq-count from HTSeq package (version 0.5.3p9) (http://www-huber.embl.de/users/anders/HTSeq/)

Genes with less than 10 fragments in all the samples were removed before differential expression analysis. The differential expression between conditions was statistically assessed by R/Bioconductor package edgeR (version 2.6.10) [41]. Paired design model was used as suggested in edgeR user's guide. Genes with FDR ≤ 0.05 were called as differentially expressed.

### Tissue purity estimation

Pancreatic cancer consists of a high percentage of stromal cells. The infiltrating stromal and immune cells form the major fraction of normal cells in tumor tissue. These cells play an important role in cancer biology but may also interfere in the analysis of tumor-specific signals. To measure the fraction of stromal and immune cells in tumor samples, we applied the ESTIMATE method [37] in our data analysis.

### Validation of selected DEGs

Top DEGs were selected for validation according to the following criteria: 1) a log ratio of ≥ 5; 2) an FDR q-value <0.001; and 3) potential biological significance in PDAC. The mRNA levels of the selected genes were measured by quantitative RT-PCR using an ABI PRISM 7900HT thermocycler (Applied Biosystems, CA). Specific primers used in these experiments are listed in Supplementary Table 2. All reactions were run in triplicate. β-actin was used for the normalization of expression data, and the 2^−ΔΔCt^ method was applied [42].

IHC for protein expression was performed on formalin-fixed paraffin-embedded sections of 8 pairs of tumor and adjacent benign pancreatic tissues from patients with resected PDAC. The tissue sections were obtained from the National Cancer Institute supported Human Tissue Network. IHC used the ABC (avidin-biotin-peroxidase complex) method and the protein expression level was scored semi-quantitatively by multiply the staining intensity (0-3) with the percentage (0-100) of positive tumor cells (histo-score, H-score) [43].

### Pathway analysis

IPA was used to map 1,460 DEGs with a FDR q-value of <0.01 to gene ontology groups and biological pathways using the Ingenuity Knowledge Base as the reference [44]. Fisher's exact test was used to calculate a probability value to indicate the association between each gene in the list and IPA-curated pathways and biological functions. A P-value less than 0.05 was considered statistically significant overrepresentation of genes in a canonical pathway or gene ontology group (e.g., molecular and cellular functions).

## SUPPLEMENTARY MATERIALS AND TABLES








