# Supplementary material for: RNA sequencing analyses reveal novel differentially expressed genes and pathways in pancreatic cancer
Source: Oncotarget. 2017 Mar 22;8(26):42537–47. doi: 10.18632/oncotarget.16451 (PMC5522086; doi:10.18632/oncotarget.16451)
Supplement: Supplementary file 1 [file oncotarget-08-42537-s001.pdf]

RNA sequencing analyses reveal novel differentially expressed genes and pathways in pancreatic cancer

SUPPLEMENTARY TABLES

Supplementary Table 1: Characteristics of the study population

ID	Tumor cell %	Age	Sex	Tumor site	Stage	# of positive/total lymph nodes resected	Grade	CA19-9 (U/ml)*
**RNA-Seq**								
pt13	>90	58	F	head	resectable	0/4	low-moderate	56.8
pt34	70	67	M	head	resectable	0/16	low-moderate	98.1
pt43	>90	56	M	head	resectable	8/31	low-moderate	616.1
pt45	>80	62	F	body	resectable	0/10	low-moderate	13.9
pt65	70	83	M	body	resectable	0/7	moderate	120.4
pt66	80	59	F	tail	resectable	1/4	low-moderate	426.6
pt67	>90	61	F	body	resectable	0/7	low-moderate	8
pt72	80	59	F	head	resectable	0/21	moderate	75.64
pt76	70	64	M	head	resectable	1/27	low-moderate	214
pt78	80	59	F	head	resectable	1/1	low-moderate	16.26
**RT-PCR**								
pt6	40	57	M	head	resectable	0/9	moderate	29.3
pt14	25	57	M	head	resectable	NA	low-moderate	303.9
pt16	>90	59	M	head	resectable	2/4	low-moderate	590.2
pt23	20	61	M	head	resectable	10/11	moderate	25.2
pt26	40	73	F	head	resectable	5/32	low-moderate	0.8
pt29	20	69	M	head	resectable	0/21	low-moderate	24
pt33	50	68	M	body	resectable	1/6	moderate	59.4
pt42	40	52	M	head	resectable	0/14	moderate	1049.5
pt54	65	61	F	head	resectable	0/19	high-moderate	24.7
pt56	15	53	M	head	resectable	0/7	moderate	48.4
pt58	60	65	M	head	resectable	1/4	moderate	17.7
pt61	50	72	M	head	resectable	3/18	high-moderate	955.3
pt62	60	59	F	tail	resectable	0/8	moderate	1185.4
pt59	30	68	M	head	resectable	0/15	low	36.52
pt17	50	66	F	whole	resectable	27/40	moderate	238.3
pt39	60	66	M	head	resectable	0/26	high-moderate	>2060
pt47	50	62	M	head	resectable	0/10	low-moderate	>2060
pt49	30	48	M	head	resectable	1/12	low-moderate	734.8
pt70	30	45	F	head	resectable	0/21	high-moderate	394.7
pt25	70	38	F	head	resectable	0/19	moderate	1472.1

*The normal range of CA 19-9 in the blood of a healthy individual is 0-35 U/ml.

Supplementary Table 2: Specific primers for qRT-PCR

Gene name	Primer sequence (5'-3')	
β-actin	Forward	GACGACATGGAGAAAATCTG
	Reverse	ATGATCTGGGTCATCTTCTC
CDX1	Forward	GGAGAAGGAGTTTCATTACAG
	Reverse	TGCTGTTTCTTCTTGTTCAC
HOXA10	Forward	CTCCCCTTCATAACAGAAAAAG
	Reverse	AATTGCCTTGACACATTTCC
KRT16	Forward	AAAGACTACAGTCCCTACTTC
	Reverse	TTGTCAATCTGCAAAATGGG
SERPINB5	Forward	ATGTCCTCTTCTCTCCAATC
	Reverse	ACCTGTCCAATTTCATTTGC
SI	Forward	ATAGACACCTATGAAAGAGACC
	Reverse	CATACATGAAGGGATCCAAG

Supplementary Table 3: List of differentially expressed genes with FDR <0.05

See Supplementary File 1

Supplementary Table 4: Signficant canonical pathways overrepresented by DEGs with FDR <0.01

See Supplementary File 2

Supplementary Table 5: Significant molecular functions overrepresented by DEGs with FDR<0.01

See Supplementary File 3
